# TREM2-Dependent Effects on Microglia in Alzheimer’s Disease

**DOI:** 10.3389/fnagi.2018.00202

**Published:** 2018-07-09

**Authors:** Yingyue Zhou, Tyler K. Ulland, Marco Colonna

**Affiliations:** Department of Pathology and Immunology, Washington University in St. Louis, St. Louis, MO, United States

**Keywords:** Alzheimer’s disease, microglia, TREM2, metabolism, autophagy

## Abstract

Alzheimer’s disease (AD) is a late-onset dementia characterized by the deposition of amyloid plaques and formation of neurofibrillary tangles (NFTs) which lead to neuronal loss and cognitive deficits. Abnormal protein aggregates in the AD brain are also associated with reactive microglia and astrocytes. Whether this glial response is beneficial or detrimental in AD pathology is under debate. Microglia are the resident innate immune cells in the central nervous system (CNS) that survey the surrounding environment. Genome-wide association studies (GWAS) have identified the R47H variant of triggering receptor expressed on myeloid cell 2 (TREM2) as a risk factor for late-onset AD (LOAD) with an odds ratio of 4.5. TREM2 is an immunoreceptor primarily present on microglia in the CNS that binds to polyanionic molecules. The transmembrane domain of TREM2 signals through DAP12, an adaptor protein that contains an immunoreceptor tyrosine-based activation motif (ITAM), which mediates TREM2 signaling and promotes microglial activation and survival. In mouse models of AD, *Trem2* haplodeficiency and deficiency lead to reduced microglial clustering around amyloid β (Aβ) plaques, suggesting TREM2 is required for plaque-associated microglial responses. Recently, TREM2 has been shown to enhance microglial metabolism through the mammalian target of rapamycin (mTOR) pathway. Although aberrant metabolism has long been associated with AD, not much was known regarding how metabolism in microglia might affect disease progression. In this review, we discuss the role of TREM2 and metabolism in AD pathology, highlighting how TREM2-mediated microglial metabolism modulates AD pathogenesis.

## Alzheimer’s Disease and Genetic Risk Factors

Alzheimer’s disease (AD), the most common form of dementia, is a progressive neurodegenerative disorder clinically distinguished by loss of memory and deficits in cognitive functions. Histologically, the hallmarks of AD are aggregation and accumulation of extracellular β amyloid (Aβ) plaques and intracellular tau protein neurofibrillary tangles (NFTs), which results in extensive neuronal death (Holtzman et al., 2011). Plaque forming Aβ peptides are derived from amyloid precursor proteins (APP) that are sequentially cleaved by β-secretase and γ-secretase (Holtzman et al., 2011). On the other hand, hyperphosphorylated, aggregated microtubule-binding protein tau dissociates from microtubules and forms NFT. Accumulation of Aβ plaques precedes tau-mediated neuronal dysfunction and cognitive decline in both autosomal dominant and late-onset AD (LOAD) patients (Jack et al., 2010; Bateman et al., 2012). Whether tau pathology is independent or downstream of Aβ remains elusive. Abnormal protein aggregates in the AD brain are also associated with a glial response, which includes activation and recruitment of microglia and astrocytes to amyloid plaques (Sastre et al., 2006; Ransohoff, 2016). The impact of AD-associated gliosis remains a topic of extensive research.

Aging is the greatest risk factor for sporadic AD. Besides that, genetic, epigenetic, and environmental factors all contribute to the complexity of AD. Mutations in *APP*, presenilin 1 (*PSEN1*), and presenilin 2 (*PSEN2*) cause a rare form of AD that occurs in an autosomal dominant fashion (Bertram et al., 2010). Patients with familial AD develop symptoms as early as their 20 s or 30 s. Mutations in *APP, PSEN1* and* PSEN2* cause increased total Aβ level or an increased ratio of Aβ42 to Aβ40, leading to familial AD (Takasugi et al., 2003). The genetics of the more common LOAD is more complex. Many genetic risk factors have been implicated in increasing susceptibility for LOAD, among which apolipoprotein E (*APOE*) confers the highest odds ratio. One copy of the ε4 allele of *APOE* increases the risk of AD by ~4 fold (Strittmatter et al., 1993), and individuals carrying two alleles of *APOE*4 have a risk of developing AD 12-fold higher than individuals with two copies of *APOE*3. In contrast, the *APOE2* allele is neuroprotective and reduces risk of AD by 50% compared with *APOE*3 (Bertram et al., 2007). Genome-wide association studies (GWAS) have identified additional genes associated with AD. Multiple variants associated with an increased risk of developing LOAD are in genes related to immune functions, including triggering receptor expressed on myeloid cells 2 *(TREM2), CD33, CR1, EPHA1* and* ABCA7* (Hollingworth et al., 2011; Naj et al., 2011; Lambert et al., 2013). Notably, multiple human heterozygous rare variants in TREM2 were found with high risks of LOAD (Guerreiro et al., 2013; Jonsson et al., 2013). The most common variant within TREM2, rs75932628, encoding an arginine to histidine at position 47 (R47H) that imparts a partial loss of function, increases the risk for developing LOAD by 4-fold (Guerreiro et al., 2013; Jonsson et al., 2013). Other TREM2 variants, including R62H, D87N, T96K, E151K, H157Y and L211P, have been associated with LOAD, although their functional effects vary and the impacts on TREM2 signaling require further investigations (Guerreiro et al., 2013; Jin et al., 2014; Song et al., 2017). Altogether, these genetic studies highlighted the important role of microglia in regulating AD progression.

## Microglia and AD

Microglia are the resident innate immune cells in the central nervous system (CNS) that account for ~10%–15% of cells. Microglia are yolk sac-derived and represent a self-renewing population that requires colony-stimulating factor 1 receptor (CSF1R) signaling for development and survival (Ginhoux et al., 2010; Wang et al., 2012; Elmore et al., 2014). Besides their function in brain immunosurveillance, microglia play an important role in brain development and synaptic plasticity by constantly surveying the surroundings. In steady state, microglia engulf synapses through the complement pathway, which is essential for synaptic connectivity and normal brain development (Stevens et al., 2007; Schafer et al., 2012; Hong et al., 2016). Cognitively, mice depleted of microglia show defective learning and memory formation abilities (Parkhurst et al., 2013).

The exact role microglia play in AD is not completely clear. *In vitro*, Aβ oligomers can induce the production of proinflammatory cytokines such as interleukin 1 beta (IL-1β) and tumor necrosis factor alpha (TNFα) in microglia primed by LPS or IFNγ, as well as trigger reactive oxygen species (ROS) and nitric oxide (NO) production (Meda et al., 1995; Parajuli et al., 2013), leading to the hypothesis that microglia are neurotoxic and contribute to a chronic neuroinflammatory environment in neurodegeneration. Consistent with the idea, knockout of the NLRP3 inflammasome pathway in APP/PS1 mice skews microglia to anti-inflammatory states and protects the mice from memory loss (Heneka et al., 2013). Additionally, Aβ deposition induces inflammasome-dependent ASC specks formation in microglia, which in turn seed Aβ oligomers and aggregates and increase Aβ pathology in a feed forward loop. This seeding is absent in ASC-deficient mice (Venegas et al., 2017), suggesting that microglia facilitate plaque formation and play a detrimental role in AD pathology. In contrast to these findings, several studies support a neuroprotective role of microglia. One striking feature of microglia in both AD mouse models and AD patients is that they cluster around plaques (Ulrich et al., 2014; Condello et al., 2015; Jay et al., 2015; Wang et al., 2015; Yuan et al., 2016), providing a protective barrier between neurons and Aβ and thus preventing neuronal dystrophy. Furthermore, primary microglia cultures are able to phagocytose Aβ complexed with apolipoproteins (Yeh et al., 2016). However, in transiently microglia depleted AD mice, the overall Aβ level is not affected, compared to wild-type (WT) AD controls (Spangenberg et al., 2016), suggesting microglia’s dispensable role in Aβ phagocytosis, which might be compensated by astrocytes.

Single-cell RNA sequencing analysis has allowed finer characterization of disease-associated microglia (DAM; also defined as microglial neurodegenerative phenotype (MGnD)), which localize to plaques in AD mouse models and are also found in other neurodegenerative models, namely amyotrophic lateral sclerosis (ALS) and experimental autoimmune encephalomyelitis (EAE; Keren-Shaul et al., 2017; Krasemann et al., 2017). DAM downregulate homeostatic microglial genes such as *P2ry12*, *Tmem119* and *Cx3cr1*, while inducing the expression of several AD associated activation markers, such as *Apoe*, *Tyrobp* and *Trem2* (Keren-Shaul et al., 2017; Krasemann et al., 2017). Keren-Shaul et al. (2017) proposed that TREM2 is required for induction of fully activated DAM, which is preceded by an intermediate state of microglial activation initiated in a TREM2-independent manner. On the other hand, Krasemann et al. (2017) showed that induction of MGnD can be initiated by phagocytosis of apoptotic neurons and is mediated by TREM2-induced expression of ApoE and miR-155. TREM2 is a critical regulator of DAM activation yet the exact role of TREM2 in this process needs further investigations.

## Functions of TREM2

**Figure 1:**
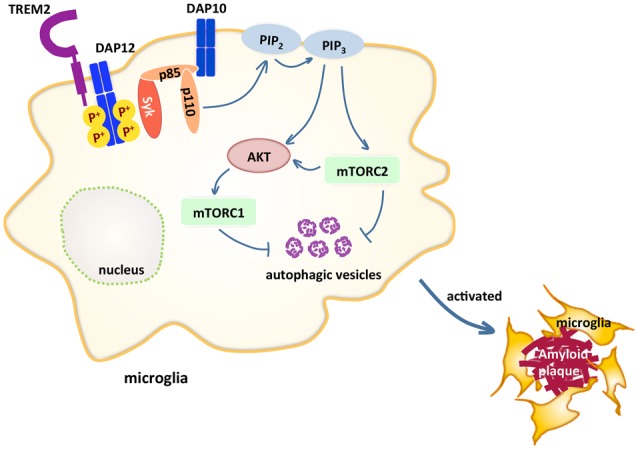
Triggering receptor expressed on myeloid cells 2 (TREM2) maintains microglial metabolism through the mammalian target of rapamycin (mTOR) pathway in Alzheimer’s disease (AD). TREM2 pairs with DAP12 through charge interactions in the transmembrane domain. Upon TREM2 ligand binding, DAP12 gets phosphorylated and recruits spleen tyrosine kinase (SYK), which initiates a cascade of signaling events, including phosphoinositide 3-kinase (PI3K) activation, which is composed of p85 and p110. The recruitment of p85 requires DAP10. One of the PI3K downstream targets AKT then activates mammalian target of rapamycin complex 1 (mTORC1) and mTORC2, which inhibits autophagy. These signaling events maintain microglia at high energy states so that in AD models, microglia are able to cluster around amyloid plaques.

TREM2 is an immunoglobin (Ig) superfamily receptor present on various cells of the myeloid lineage including CNS microglia, bone osteoclasts, alveolar and peritoneal macrophages (Colonna and Wang, 2016). TREM2 consists of an extracellular V-type Ig-like domain, a transmembrane domain, a stalk region that connects the two and a short cytoplasmic tail. TREM2 binds to polyanionic molecules such as bacterial lipopolysaccharide (LPS; Daws et al., 2003), phospholipids (Wang et al., 2015), lipoproteins such as HDL and LDL (Song et al., 2017), which form complexes with APOE and APOJ (Atagi et al., 2015; Yeh et al., 2016) and apoptotic neurons, and signals through DAP12 (TYROBP; Figure 1). DAP12 is an adaptor protein that contains immunoreceptor tyrosine-based activation motifs (ITAMs), which function as docking sites for protein kinases. Upon TREM2 ligand binding, the ITAMs of DAP12 get phosphorylated and recruit spleen tyrosine kinase (SYK), which initiates protein tyrosine kinase phosphorylation, phosphoinositide 3-kinase (PI3K) activation, efflux of Ca^2+^ and mitogen-activated protein kinase (MAPK) activation. One report showed that DAP10, an adaptor closely related to DAP12, is required for the recruitment of the p85 subunit of PI3K to DAP12 (Peng et al., 2010; Figure 1). The triggering of kinase cascades by TREM2 activation promotes microglial survival, proliferation and leads to rearrangement of actin cytoskeleton. Lack of *Trem2* impairs proliferation of osteoclast precursors (Otero et al., 2012) and *Trem2*^−/−^ microglia or macrophages are less viable under stress (Wang et al., 2015; Wu et al., 2015). *In vitro*, several studies have suggested that TREM2 may be a phagocytic receptor that mediates phagocytosis of apoptotic neurons (Takahashi et al., 2005; N’Diaye et al., 2009) or lipidated Aβ (Yeh et al., 2016). TREM2 mutations Y38C and T66M that reduce cell surface TREM2 expression impair phagocytosis (Kleinberger et al., 2014). Migration of microglia towards injected apoptotic neurons was also attenuated in *Trem2*^−/−^ mice (Mazaheri et al., 2017). In addition, TREM2 also modulates inflammation. In *Trem2*^−/−^ or *Dap12*^−/−^ macrophages stimulated with low amounts of toll-like receptor (TLR) agonists, the level of inflammatory cytokines produced, such as TNFα and IL-6, is significantly increased (Hamerman et al., 2006; Turnbull et al., 2006), indicating TREM2 modulates TLR-mediated inflammatory responses but only in response to low levels of TLR stimulation. Furthermore, Aβ binds to TREM2 and activates TREM2 signaling pathway (Lessard et al., 2018; Zhao et al., 2018). Whether TREM2 binding to Aβ affects inflammatory responses in microglia needs further studies, since very few changes in cytokine production after Aβ stimulation alone were detected (Zhao et al., 2018).

Membrane bound full-length TREM2 undergoes sequential protease cleavages by a disintegrin and metalloproteases (ADAMs) and γ-secretase, which produces soluble TREM2 (sTREM2; Wunderlich et al., 2013; Kleinberger et al., 2014). In AD patients, sTREM2 levels increase in the cerebrospinal fluid (CSF) and correlate with the levels of tau in CSF (Suárez-Calvet et al., 2016), indicating sTREM2 as a biomarker for AD. It is possible that sTREM2 acts as a decoy receptor that inhibits full length membrane-bound TREM2 from binding to its ligands. Recently, two groups suggested sTREM2 increases cell viability (Wu et al., 2015; Zhong et al., 2017). However, a receptor responsible for activating downstream survival signals was not identified and thus the physiological function of sTREM2 remains elusive.

## TREM2 and AD Pathology

The importance of TREM2 in the CNS was first highlighted by the discovery of Nasu-Hakola disease (NHD), a rare autosomal recessive disorder presented with an early onset dementia. Homozygous loss-of-function mutations in either TREM2 (such as Q33X, Y38C and T66M) or DAP12 lead to NHD and FTD-like syndrome (Bianchin et al., 2006; Guerreiro et al., 2013). More recent studies have associated TREM2 with increased risk of several neurodegenerative diseases, including AD (Guerreiro et al., 2013; Jonsson et al., 2013), Parkinson’s Disease (Rayaprolu et al., 2013), frontotemporal dementia (Thelen et al., 2014) and ALS (Cady et al., 2014).

Mechanisms of TREM2-related neurodegeneration have been intensively investigated. Many studies have shown that microglia in mouse models and patients with AD upregulate TREM2 expression (Frank et al., 2008; Lue et al., 2015; Wang et al., 2015), suggesting that upregulation of TREM2 may be associated with AD progression. Moreover, TREM2 plays a prominent role in driving microgliosis in AD mouse models and patients. In AD mice expressing WT *Trem2*, microglia cluster around plaques, providing a barrier to the surrounding neurons (Ulrich et al., 2014; Jay et al., 2015; Wang et al., 2015, 2016; Yuan et al., 2016). On the contrary, the number of plaque-associated microglia is significantly reduced in *Trem2* deficient AD mice, and the effect is *Trem2* gene dose-dependent (Jay et al., 2015; Wang et al., 2015). In both transgenic mice expressing the human TREM2 R47H or patients with the R47H variant, a similar reduction in microgliosis is observed (Yuan et al., 2016; Song et al., 2018), supporting that R47H is a loss-of-function mutation. Consistently, a larger number of dystrophic neurites accumulate around plaques in *Trem2*^−/−^ 5xFAD mice, compared to WT *Trem2* 5xFAD. The observations of plaque-associated microglia are consistent with the identification of DAM (Keren-Shaul et al., 2017; Krasemann et al., 2017). As DAM are phagocytic, one would hypothesize that in *Trem2*^−/−^ AD mice, lack of DAM would lead to increased Aβ burden. However, observations on plaque load in *Trem2*-deficiency AD mice are inconsistent (Ulrich et al., 2014; Jay et al., 2015; Wang et al., 2015), which is likely due to different mouse models used or timing at which the analyses were done (Jay et al., 2017). Despite discrepancies on plaque load, changes in plaque morphology have been reported by several groups. In *Trem2*^−/−^ 5xFAD mice, amyloid plaques appear with loosely packed cores and more diffuse structures extending outward (Wang et al., 2016; Yuan et al., 2016), which is also observed in R47H carriers (Yuan et al., 2016), suggesting that TREM2-mediated microglia-plaque interaction may be critical for compacting amyloid fibrils. Whether the changes in plaque structure are relevant to the role of TREM2 in AD is uncertain, since diffuse non-fibrillar plaques also occur in cognitively normal individuals (Morris et al., 2014). More studies are needed to investigate if compositions of plaques are different. Besides, whether the increased neuritic dystrophy in *Trem2* deficient mice is due to impaired clearance of damaged neurites by *Trem2*^−/−^ microglia or the result of elevated damage by loosely compacted plaques also remains unclear. Collectively, these studies indicate that TREM2 signaling promotes microglial responses to Aβ in AD.

The effects of TREM2 on tau-driven AD models have not been intensely investigated. Recently, two groups that studied tau models showed inconsistent results. In the PS19 tau transgenic mice that express a human tau with the P301S mutation, lack of *Trem2* leads to less brain atrophy but no change in tau phosphorylation or aggregation. These tau mice lacking *Trem2* show reduced microgliosis and decreased microglial activation at 9-month old (Leyns et al., 2017). In contrast, in another study using a mouse model expressing the full-length human tau gene, *Trem2* deficiency resulted in elevated hyperphosphorylation and aggregation of tau (Bemiller et al., 2017). As with Aβ models, controversy on effects of TREM2 in tau pathology remains. The relationship of Aβ and tau and how it is affected by TREM2 are interesting topics for future studies.

## TREM2 and Metabolism

Metabolic dysfunctions have long been associated with AD, while most studies focused on neuronal metabolisms. Our group and others have recently linked defective microglial function to metabolism in dementia. Kleinberger et al. (2017) demonstrated that cerebral metabolic rates of glucose slowed down as shown by reduced FDG-uPET signal in a Trem2 T66M knock-in mouse model of NHD. The reduced glucose usage could be due to defective microglial function that impairs the metabolic states of the brain, hinting that dysfunctional TREM2 might alter the brain metabolism and thus promote pathogenesis (Kleinberger et al., 2017).

In another study, it was shown that *Trem2*-deficient bone marrow-derived macrophages exhibit a defective energetic and anabolic state, which is further exacerbated by stress, such as CSF1 reduction (Ulland et al., 2017). This metabolic defect is a result of reduced mammalian target of rapamycin (mTOR) signaling, which is ameliorated by activation of Dectin-1, a receptor that activates downstream PI3K and mTOR independent of TREM2, or cyclocreatine, a creatine analog that restores ATP level. Impaired mTOR signaling was also observed in microglia sorted from *Trem2*^−/−^ 5xFAD mice. These results suggest that TREM2 maintains microglia at high metabolic states through enhanced activation of the mTOR pathway (Figure 1). Furthermore, increased autophagy is detected in *Trem2*-deficient microglia and in AD patients carrying one allele of the R47H or R62H variant of TREM2, suggesting microglia attempt to compensate the mTOR defects with autophagy as a survival mechanism.

## Autophagy and AD

Autophagy is a self-eating process where cells clear misfolded proteins and damaged organelles as a housekeeping mechanism and as a survival mechanism during stress and starvation. The formation of an autophagophore, an isolation membrane that initiates autophagy and becomes the autophagosome later, requires the ULK complex and the class III PI-3 kinase (PI3K) complex composed of Vps34, Beclin-1, p150 and ATG14. The ULK complex is inactivated by mammalian target of rapamycin complex 1 (mTORC1) and positively regulated by AMP-activated protein kinase (AMPK). Activation of ULK and PI3K complexes recruit additional autophagy related (ATG) proteins to drive autophagosome nucleation. Then the ATG12-ATG5-ATG16 complex is recruited to facilitate lipidation of microtubule-associated protein 1 light chain 3 (LC3), resulting in the conversion of LC3-I to LC3-II, as the isolation membrane expands to form the autophagosome (Kabeya et al., 2000). In mammalian cells, only LC3-II remains on autophagosome membranes until after their fusion with lysosomes, allowing it to be a marker for autophagy (Mizushima et al., 2010).

Autophagy has been associated with AD. The levels of autophagy protein Beclin 1 are decreased in AD patients but not in AD mouse models (Pickford et al., 2008), pointing to the possibility that Beclin 1 reduction occurs upstream of amyloid pathology. Genetic ablation of one copy of *Beclin1* reduces autophagy in cultured primary neurons and increases Aβ deposition and neuronal loss in an AD model. On the other hand, an increase of CD68 immunoreactivity, an activation marker, with the absence of change in Iba1 marks microglial activation in *Beclin 1*^+/−^ AD mice without affecting microglial number (Pickford et al., 2008). These findings indicate that neuronal autophagy plays a protective role in AD progression. In line with this, overexpression of another autophagy gene, p62, by adeno-associated virus (AAV) infection in neurons improves cognitive functions and reduces Aβ pathology in an autophagy-mediated manner (Caccamo et al., 2017). Pharmaceutically, feeding mice with rapamycin, an inhibitor of the mTOR pathway, induces autophagy in two mouse models of AD but not in non-AD littermate controls, suggesting high Aβ level is a trigger of autophagy. This rapamycin-mediated mTOR inhibition leads to a reduction of Aβ42 level in the hippocampus and an amelioration in memory deficits by increasing autophagy (Caccamo et al., 2010; Spilman et al., 2010). Although all these studies suggest a protective function of autophagy, whether this effect is only mediated by neurons or if glia also play a role is unclear.

Rather, our study demonstrated a role of TREM2 in attenuating microglial autophagy (Ulland et al., 2017). Compared to control, microglia lacking TREM2 contain more autophagic vesicles as shown by electron microscopy. Increased autophagy in *Trem2*-deficient microglia is a result of reduced mTOR signaling in response to a low metabolic state of microglia and this increase in autophagy is not sufficient to rescue microglia from dying under stress, such as neuroinflammation. Interestingly, dietary supplementation with cyclocreatine rescues *Trem2*-deficient microglia from autophagy and partially corrects the defect in microglial clustering in *Trem2*^−/−^ 5xFAD. These results suggest that TREM2 sustains microglial metabolism thus allowing them to function properly. This study links TREM2 to metabolic states of microglia and allows us to revisit the role of TREM2 and microglia in AD pathology.

## Future Perspectives

The role of microglia in neurodegeneration has been hotly debated while no concluding agreement has been made. Such an agreement is difficult to achieve as different groups using similar models show different results, perhaps due to microbiota. Notably, studies on germ-free mice in AD models are currently being conducted. As TREM2 is required to turn homeostatic microglia into activated microglia, whether these activated microglia acquire a unique metabolic state is an intriguing topic. It remains unclear what exact role TREM2 plays. Does it sense changes in environment or just provide a tonic signal to keep microglia ready to go? Further studies are needed to distinguish the two possibilities. Defining the ligands of TREM2 *in vivo* and how it is activated will help answer the question. Microglia can be a double-edged sword. Deciphering the role of DAM will advance our therapeutic approaches in neurodegenerative diseases.

## Author Contributions

YZ drafted the manuscript. TU and MC reviewed and edited the manuscript.

## Conflict of Interest Statement

The authors declare that the research was conducted in the absence of any commercial or financial relationships that could be construed as a potential conflict of interest.
